# Shared stressful experiences affect social proximity in Merino sheep

**DOI:** 10.1098/rsbl.2022.0396

**Published:** 2023-02-08

**Authors:** Hamideh Keshavarzi, Caroline Lee, Tim R. Dyall, Mark Johnson, Dana L. M. Campbell

**Affiliations:** ^1^ Agriculture and Food, Commonwealth Scientific and Industrial Research Organisation (CSIRO), Armidale, New South Wales 2350, Australia; ^2^ Data61, Commonwealth Scientific and Industrial Research Organisation (CSIRO), Marsfield, New South Wales 2122, Australia

**Keywords:** shared experience, real-time kinematic (RTK) devices, social bonds, familiar

## Abstract

While it is well established that humans develop stronger relationship bonds when they share stressful experiences, there is little known on how shared stressful experiences may influence relationship bonding in animals. Here, we present a study looking at social proximity between individuals in small groups of Merino ewes following a shared stressful experience compared with control sheep that were not exposed to stress. Some sheep were familiar to each other. Analyses of social proximity using real-time-kinematic Global Navigation Satellite System (GNSS) on-animal devices showed sheep preferred to be closest to familiar individuals, but across the study duration they also developed a preference for the individuals they shared the stressful experience with, relative to their proximity to control individuals. These results contribute to limited research on what factors may instigate the development of bonds between unfamiliar sheep. Between-individual bonds may develop as a means of socially mediated stress buffering. Social bonding following a shared stressful experience aligns with human social relationships and increases our understanding of how animals perceive their conspecifics in relation to stressful environmental change.

## 1. Introduction

Group-living animals across many different species will show non-reproductive social bonds with specific individuals [1,2]. These bonds can be evidenced by increased time in proximity, behavioural synchrony or socially connective behaviours such as allogrooming [1,2]. While the origins of a social bond developed from a reproductive or kin-based association may be relatively straightforward, other mechanisms of social bond development are less clear [3]. Forced time spent together has resulted in subsequent increased allogrooming in captive common vampire bats [4] and randomly assigned college roommates will develop lasting friendships [5,6]. Time spent together sharing a specific experience may also create new social relationships. Supporting evidence in humans is well-established where shared experiences, both positive and negative, such as singing [7] or military-related trauma [8,9], will result in bond formation. Comparatively, there is more limited evidence in non-human animals [3]. A shared experience of watching a video together increased social proximity between individual great apes [10] and a perceived shared risk of predation in guppies resulted in more stable and more differentiated social proximities in the group [11]. Further studies across other non-human animals would strengthen our understanding of whether shared positive or negative experiences can lead to affiliative social bond development [3].

A shared negative experience may increase social association via mechanisms of survival-driven cooperation [12,13], or stress relief [14]. The benefits of social support as a stress-buffering mechanism have been documented across many non-human animal species [15–18]. Social partners can reduce physiological and/or behavioural stress responses to threats [19], to intraspecific aggression [20], moving to new environments [21] and can enhance recovery after illness/injury [22]. Thus, increased social affiliation as a result of negative, stressful experiences could have implications across taxa for greater resilience and adaptation. For example, rhesus macaques showed increased social affiliations and greater grooming investments in new relationships during reestablishment following a natural hurricane disaster [23].

Alternative to shared negative experiences forging new social bonds, individuals may seek the comfort of previously established social relationships. Across taxa, unfamiliar conspecifics can represent stressful threats to individuals and their resources [14,20]. Thus, when given a choice during experimental testing, several animal species will show a preference for familiar over unfamiliar individuals following an induced stressor, or familiar conspecifics will be more effective at attenuating physiological stress responses [15,19,24]. In sheep (*Ovis aries*), familiar individuals are recognized and preferred over unfamiliar conspecifics [25,26] and they will have a stress buffering impact [25,27]. However, social isolation is a significant stressor in this gregarious species [28,29], driving establishment of social connections. A shared negative experience may accelerate the creation of social bonds in unfamiliar groups of individuals.

The objective of this study was to measure spatial proximity to determine if a shared stressful experience would create new social bonds in unfamiliar sheep relative to unfamiliar control or familiar individuals in a group setting. It was predicted that sheep would prefer the familiar individuals but that the shared negative stress would lead to closer proximity between the treatment animals than with control sheep.

## 2. Materials and methods

### (a) Animals and treatment protocol

Seventy intact, non-pregnant and cycling Merino ewes (3 to 6 years of age) across five sequential test cohorts were used in the study from 24 May to 18 June 2021 (autumn/winter). As part of farm management, the ewes would have been mated late May, but this did not occur as they were part of the study. The animals were selected from five established farm flocks (119–400 sheep/flock) to have a range of sheep that were predominantly unfamiliar with each other. Two weeks prior to testing, 14 sheep (test animals plus spare companions) were selected from each of the five larger flocks (*n* = 70) and moved to five smaller separate holding paddocks with grazing pasture and drinking water available. An empty paddock was left in between each holding paddock to ensure unfamiliarity between sheep flocks prior to being mixed into testing cohorts. The sheep remained in these smaller paddocks until they had finished their testing, after which they were returned to a separate holding paddock, distant from any remaining testing groups. Cohorts of 10 sheep were tested sequentially, thus, as testing progressed, the number of sheep in the holding paddocks reduced (necessitating spare companion animals). Furthermore, the sheep tested in the later cohorts had a longer duration of time together within the smaller holding paddocks (maximum 5 weeks together). For each test cohort, on day 1, two sheep from each of the five flocks were randomly selected in the morning (*n* = 10 sheep per test cohort). One sheep per flock (five in total) were trailered to yards for the induced stress treatment, and the other five sheep (control treatment) were walked down to be adjacent to the main testing paddock where they remained until the afternoon when the corresponding stressor treatment had been completed.

The stress treatment comprised a range of stressors applied to all five sheep on day 1 across a period of approximately 4.5 h, out of sight of the control sheep. The selected stressors were intermittent stressful activities [30–33] that would occur as part of routine farm management but were compressed over a shorter time duration to create a shared negative experience (table 1). A stressor was applied every 45 min as follows: trailer transport for 15 min, individual restraint for 2 min (the handler's body firmly held against the sheep onto the side of the pen), moved through the yards with a dog for 5 min, simulated crutching (removing wool from around the tail and between the rear legs with a shearing device) for 2 min, followed by a repeat of trailer transport, restraint and yard moving (table 1). The individual animal stressors of restraint and simulated crutching were conducted sequentially across all five treatment sheep in the presence or proximity respectively, of the other sheep in the group. The timeline was determined based on typical cortisol profiles where peak cortisol occurs approximately 10–15 min after a stressor commences and then slowly declines over the next 60 min [31,32,34]. Continuing the stressors ensured a negative experience was being induced while not resulting in any physical sheep harm, although no physiological measures of stress were taken on the sheep.

**Table 1.** Summary of the induced stress treatment.

start time	stressor	duration (min)
10.00	trailer transport (group)	15
10.45	restraint: tipped and held still by human	2 min per sheep
11.30	moving as a mob through yards with a dog	5
12.15	simulated crutching (wool removal around legs/tail) with electric handpiece	2 min per sheep
13.00	trailer transport (group)	15
13.45	restraint: tipped and held still by human	2 min per sheep
14.30	moving as a mob through yards with a dog	5

### (b) Social distance and network

#### (i) Real-time kinematic rovers and positional data collection

For each cohort, on day 1 following the stress treatment, all animals had an RTK (real-time kinematic) rover fitted on their backs (just behind the shoulder) via a dog harness (Comfy Harness, size 8, 84–120 cm, Company of Animals, Surrey, UK) to record the Global Navigation Satellite System (GNSS) positional data (see electronic supplementary material, figure S1). The device uses a combination of GNSS and RTK that greatly increases the accuracy of the GNSS signals by using a base station that wirelessly sends positional corrections to the receiver on the sheep. Further details on the validation and use of this device are described in [35,36]. Following device fitting around 15.00, all 10 animals (stressed and control) were placed into a paddock approximately 100 m × 70 m in size. Positional data were collected by the RTK rovers at a 1 s sampling rate for each cohort across 4 days. To facilitate visual checking, each sheep was numbered with coloured sheep wool marker (Heiniger Shearing Supplies, Bibra Lake, WA, Australia) that matched different coloured antennas on the RTK rovers. Some RTK rovers slipped to the side during the testing days, so all animals were brought into the yards to fix them before being placed back into the test paddock. All nine occurrences of readjustment took 14 min on average and recorded data at these times were removed from the analyses. An MEA weather station (Green Brain, 41 Vine Street, Magill, SA 5072, Australia) on site recorded ambient temperature (°C), relative humidity (%), solar radiation (W m^−2^) and wind speed (km h^−1^) every 15 min across the trial duration. See electronic supplementary material, table S1 for the averages of these four weather parameters for each cohort.

#### (ii) Calculating the social distance between animals

To measure the distances between individual sheep in each group, GNSS data collected per second for all five cohorts across 4 days were used. The performance of the 10 devices across 4 days was good except for partial failures of two devices in cohort 4 resulting in no data for these devices from the second day of study onwards. Daytime only data where visual contact between sheep was possible were used to estimate distances. The distances between all animals within a cohort were estimated using the function of *edge_dist* of the *spatsoc* package [37] in R software while considering a threshold distance of 3 m, 5 m and 10 m within a time interval of 15 min. In previous research, threshold distances of 2 m and 30 m were used to calculate social distances where 30 m encompassed all interactions but 2 m resulted in some missing data due to sheep not being at that proximity [36]. Thus, a range of threshold distances were selected in the current study to ensure minimal missing data and confirmation of social distance patterns across both near and farther distances. The final dataset included a total of 10 811, 12 065 and 13 128 observation points for all cohorts during 4 test days for the threshold distances of up to 3 m, 5 m and 10 m, respectively. Based on the objectives of the study, there were three groups of animals to measure distances between, including stress (S-S), control (C-C) and familiar (F-F). The familiar animals (F-F) were the pairs that originated from the same flocks where one individual from each pair was exposed to the stress treatment, the other was a control individual.

ANOVAs were then used to analyse differences in distances between the three groups of S-S, C-C and F-F at each threshold distance separately. Initial datasets were checked via *qqplot* in R to confirm no outliers were present. Data were tested for normality using visual assessment of Q–Q plots and the Shapiro–Wilk test and then orderNorm transformed to approach normality. The analysis was performed for all cohorts combined considering treatment (stress, control and familiar) and day (1, 2, 3 and 4) as fixed effects, including their interaction with time interval (15 min) and weather data (the average of temperature (°C), humidity (%), solar radiation (Wm^−2^) and wind speed (km h^−1^)) for each 15 min time interval as covariates. For all analyses, non-significant effects (*p* > 0.05) were removed from the final model. The *lsmeans* function in the *lsmeans* package [38] was used to estimate the least squares means (LSM) for all analyses. Where significant interaction effects were present, *post-hoc* Tukey's tests were used to determine differences (*p* < 0.05) between groups using the conditioning symbol to restrict the comparison to differences between group distances across time [39]. Mean distance differences between groups at each threshold distance were calculated on the raw data.

## 3. Results

Across all cohorts combined, there was a significant interaction between treatment and day for the three threshold distances (all *p* ≤ 0.04, table 2). Across all study days, familiar sheep preferred to stay 0.13 m, 0.26 m and 0.60 m closer to each other relative to the control sheep at threshold distances of up to 3 m, 5 m and 10 m respectively (all *p* < 0.05, table 2). The stress treatment group were 0.05 m, 0.16 m and 0.36 m closer to each other than to control sheep at threshold distances of up to 3 m, 5 m and 10 m respectively on day 3, and 0.33 m closer on day 4 at the 10 m threshold difference (all *p* < 0.05, table 2). Across treatments, on average, individuals were 0.06 m, 0.18 m and 0.81 m closer on the last day of the experiment at the threshold distances of up to 3 m, 5 m and 10 m respectively (table 2).

**Table 2.** Mean (and standard error of the mean) of the distance between animals with different treatments by day for three different threshold distances.

threshold distance/ day	treatments^a,b,c^ (mean ± SEM of distance (m))			*F*-value	*p*-value
	F-F	S-S	C-C		
up to 3 m				_(6,10796)_ = 2.15	0.04
day 1	1.84 ± 0.02^a^	2.00 ± 0.02^b^	2.03 ± 0.02^b^		
day 2	1.81 ± 0.02^a^	1.93 ± 0.01^b^	1.96 ± 0.01^b^		
day 3	1.83 ± 0.02^a^	1.89 ± 0.01^b^	1.94 ± 0.01^c^		
day 4	1.85 ± 0.02^a^	1.93 ± 0.01^b^	1.94 ± 0.01^b^		
up to 5 m				_(6,12048)_ = 3.52	<0.01
day 1	2.67 ± 0.04^a^	3.02 ± 0.05^b^	3.09 ± 0.03^b^		
day 2	2.58 ± 0.02^a^	2.79 ± 0.03^b^	2.84 ± 0.02^b^		
day 3	2.59 ± 0.02^a^	2.69 ± 0.03^b^	2.85 ± 0.02^c^		
day 4	2.56 ± 0.02^a^	2.71 ± 0.04^b^	2.77 ± 0.03^b^		
up to 10 m				_(6,13111)_ = 3.51	<0.01
day 1	3.98 ± 0.10^a^	4.86 ± 0.08^b^	4.91 ± 0.07^b^		
day 2	3.71 ± 0.06^a^	4.08 ± 0.05^b^	4.22 ± 0.04^b^		
day 3	3.63 ± 0.06^a^	3.91 ± 0.04^b^	4.27 ± 0.04^c^		
day 4	3.61 ± 0.07^a^	3.80 ± 0.05^b^	4.13 ± 0.06^c^		

^a^F = familiar, S = stress treatment, C = control group.

^b^Means with the same letter are not significantly different from each other within rows.

^c^Raw values are presented with analyses conducted on transformed data.

## 4. Discussion

Understanding how stressful experiences influence social dynamics has implications for adaptation and resilience in both captive and wild animal populations. This controlled experiment evaluated whether a shared stressful experience influenced social bonds in small groups of domestic sheep. As predicted, the sheep preferentially associated with individuals that were already familiar to them. However, over time, sheep that were exposed to stressors together showed closer proximity relative to the control group indicating the shared stress may have created a social bond.

The closer associations that developed within the shared stress group were likely a result of the stress buffering effects of social bonds evidenced across multiple animal species [15–18]. Social companions are believed to modulate the hypothalamic–pituitary–adrenal axis stress response through increased oxytocin release which lowers cortisol [15,18]. Sheep use visual cues to recognize conspecific faces [25,40] and can discriminate between conspecific olfactory cues [41]. It is thus possible that across the trial duration, sheep remembered the faces or scents of those they had experienced the stress with and had been buffered by, leading to preferential social proximity over the control animals. The differences in social proximity distances between treatment groups identified in this study were small as they were all under a 1 m distance across the three threshold distances. Measurements of this smaller magnitude were facilitated by the high geo-spatial accuracy of the novel RTK rovers. These differences in distances were interpreted as biologically relevant as it is closer proximity interactions or nearest neighbours during grazing that are indicative of social relationships within sheep flocks [42–44].

In this study, the familiar individuals were the most preferred. This aligns with previous sheep research [42,45,46] as well as across multiple other species [15,24,47] as unfamiliar conspecifics can often represent a threat to territories, food resources, offspring or mates [48]. However, across species, even unfamiliar social companions can provide social buffering effects when compared to social isolation [27,49,50]. The available number of separate sheep flocks located on the research farm for this study necessitated familiarity between some individuals. It is possible that the proximity between individuals in the shared stress treatment group may have been closer across the whole study duration if no familiar individuals were present. Furthermore, no invasive physiological measures of stress were taken in the current study to confirm cortisol elevation during the imposed stressors. Future studies could assess negative stressors of varying magnitudes and assess if the degree of cortisol elevation impacts the strength of new bond formation. Another factor that may have influenced sheep behaviour in our study was that while the ewes were non-pregnant, they may have been at different stages of the reproductive cycle. Ewe activity and speed of movement has been reported to increase during the oestrus period [51], however, the implications of this on social dynamics of sheep are not known. The physiological and behavioural stress response can also differ depending on the reproductive phase of ewes [52]. Future studies should ensure reproductive status of ewes is similar through hormonal means of synchronization. While the shared negative experience led to increased proximity, shared experiences can also be positive. Events such as food sharing could also lead to preferential bonds between unfamiliar individuals [53,54] and this warrants assessment in a controlled setting.

This study adds to the limited knowledge on factors that initiate bonds between unfamiliar individuals. Social interactions between conspecifics following negative stressful experiences could dictate population resilience through social buffering of stress where strong social connections can predict longevity [55]. This may have widespread implications under increased human-related interference, habitat fragmentation, and natural disasters. Determination of factors that can establish or accelerate social bonds increases our understanding of how animals perceive their conspecifics in relation to stressful environmental change.

## Data Availability

All raw data supporting this study are available in the CSIRO Data Access Portal: https://doi.org/10.25919/2qdw-fz52. The data are provided in the electronic supplementary material [56].
